# Ultrasound device as a minimally invasive approach for caries dentin removal

**DOI:** 10.1590/0103-6440202203878

**Published:** 2022-03-07

**Authors:** João Felipe Besegato, Priscila Borges Gobbo de Melo, Adilson César de Abreu Bernardi, Vanderlei Salvador Bagnato, Alessandra Nara de Souza Rastelli

**Affiliations:** 1 Department of Restorative Dentistry, School of Dentistry of Araraquara, São Paulo State University - UNESP, Araraquara-SP, Brasil; 2 Department of Biology and Health Sciences, University of Araraquara - UNIARA. Araraquara-SP, Brasil; 3 Department of Materials Science and Physics, Physics Institute of São Carlos, University of São Paulo - USP. São Carlos-SP, Brasil

**Keywords:** Dental caries, ultrasound energy, caries removal, scanning electron microscopy, minimally invasive dentistry

## Abstract

The aim of this study was to evaluate the efficacy of an ultrasound device and the dentin surface morphology after removal of the caries dentin lesions by removal rate and scanning electron microscopy (SEM). The Knoop hardness test on the bovine dentin blocks (n = 20, 4x4x2mm) was performed to standardize the samples and only those with 38 ± 2 KHN were included. The dentin blocks were submitted to induction of artificial caries lesions, using the bacterial model. Strains of *Streptococcus mutans* and *Lactobacillus acidophilus* were used for 7 days. The caries dentin lesion was removed for 1 min, according to two methods: G1 - carbide bur under low-speed rotation (control group) and G2 - ultrasound device under refrigeration. For the removal rate, the samples were weighed 3 times: T0 (before induction), T1 (after induction) and T2 (after removal). Morphology evaluation of the residual dentin surface was performed by SEM. Data normality was verified by Shapiro-Wilk test (p ≥ 0.240). T-test for independent samples was applied to evaluate the removal rate. A significance level of 5% was adopted. G2 provided lower removal rate than G1 (G1: 3.68 mg and G2 = 2.26 mg). SEM images showed different morphological characteristics between the groups. G2 showed absent of smear layer, while G1 showed a visible smear layer over the surface. We concluded that ultrasound device provides minimally invasive removal with residual dentin exhibiting open dentin tubules and no smear layer formation and no bacteria, which infer the removal of the infected tissue.

## Resumo

O objetivo deste estudo foi avaliar a eficácia de um dispositivo ultrassônico e a morfologia da superfície dentinária após a remoção da dentina cariada por meio da taxa de remoção e imagens de microscopia eletrônica de varredura (MEV). Foi realizado o teste de dureza Knoop nas amostras de dentina bovina (n = 20, 4x4x2mm) afim de padronizar as amostras e foram incluídas apenas aquelas com 38 ± 2 KHN. As amostras de dentina foram submetidas à indução de lesões artificiais de cárie, utilizando-se modelo biológico contendo cepas de *Streptococcus mutans* e *Lactobacillus acidophilus* durante por 7 dias. As lesões foram removidas por 1 min, de acordo com dois métodos: G1 - fresa esférica sob baixa rotação (grupo controle) e G2 - dispositivo ultrassônico sob refrigeração. Para a taxa de remoção, as amostras foram pesadas em três tempos: T0 (antes da indução), T1 (após a indução) e T2 (após a remoção). A avaliação morfológica da superfície residual da dentina foi realizada por MEV. A normalidade dos dados foi verificada pelo teste de Shapiro-Wilk (p ≥ 0,240). Teste T para amostras independentes foi aplicado para avaliar a taxa de remoção. Foi adotado nível de significância de 5%. G2 apresentou menor taxa de remoção que G1 (G1: 3,68 mg e G2 = 2,26 mg). As imagens de MEV mostraram características morfológicas diferentes entre os grupos. G2 mostrou ausência de smear layer, enquanto G1 mostrou grande quantidade de smear layer sobre a superfície. Com base neste estudo *in vitro*, o dispositivo de ultrassom promoveu remoção minimamente invasiva e dentina residual exibindo túbulos dentinários abertos e mínima formação de smear layer.

## Introduction

Due to researches leading to a better understanding of the caries process and the trend toward minimally invasive dentistry, caries excavation has become more conservative ^1^. It is well established that only the infected dentin, which is strongly infected with viable microorganisms and exhibit irreversible denaturation and disorganization should be removed ^2^ ^,^ ^3^. Otherwise, the internal layer of dentin, so called caries-affected dentin, which presents a low level of microorganism’s infection and high remineralization capacity should be preserved ^4^ ^,^ ^5^. Besides, with the development of adhesive systems and bioactive restorative materials, removal of great amounts of dental hard tissues is no longer justified ^(^ ^1^. However, dental clinicians have difficulty in distinguish these tissues and at this point, dentin excavation should be better controlled.

Usually, carious dentin is mechanically removed with excavator and/or low-speed dental burs ^6^ ^,^ ^7^. These techniques are indiscriminate and non-selective for carious tissues removal ^8^. Besides, to ensure complete elimination of the infected dentin, dental clinicians tend to include all soft, discolored, and stained dentin during excavation ^6^. This procedure often results in unnecessary removal of sound dentin or tissue with reduced mineral content and remineralization capacity, such as caries-affected dentin ^9^ ^,^ ^10^.

Sonic and ultrasonic devices are commonly used in several dental practices, such as periodontology ^11^ and endodontics ^12^. These devices belong to a conservative and alternative group so-called “micro-traumatic” tool to caries removal ^13^. In addition, it has been claimed that ultrasonic tips can be useful for precise and controlled removal of caries ^14^ and the oscillation provide by the ultrasonic energy may result in minimally invasive cavity preparation ^13^. The ultrasonic vibration can be produced by the dimension deformation of piezoceramic disks due to the switch of the electric charge, which is called the piezoelectric method ^15^. This method exhibits high efficiency of energy transfer, reducing the temperature rise and energy consumption ^15^. These characteristics of the ultrasound energy appear to contribute for minimally and precisely removal of tissues. Moreover, the harder the tissue, the easier to be cutter by ultrasound instruments. Thus, soft dentin, such as the affected dentin apparently could not be removed ^16^ ^,^ ^17^. In general, diamond tips are coupled in ultrasonic devices that are projected for several indications. However, there is no specific device for caries dentin removal, which is capable to provide specific parameters for a selecting removal.

The caries removal method chosen by the dental clinician plays an important role in the following clinical steps of the restorative procedure. Different removal techniques yield different dentin surface characteristics which may affect the bonding of the adhesive systems used ^18^ ^,^ ^19^. The quality of bonding between dentin surface and restorative material depends on the presence of smear layer formed after removal and the hybrid layer resulted from the interaction between adhesive systems and etched surface ^20^. To evaluate the residual surface, scanning electron microscopy has been used ^18^.

This paper aimed to evaluate *in vitro* the dentin removal effectiveness using an ultrasound device and the residual dentin surface morphology after removal of artificial caries lesions by the removal rate and SEM images, respectively. The null hypothesis tested were: I - there is no difference on the removal rate of dentin between the caries removal method employed; II - there is no difference on the residual dentin surface morphology regardless of the caries removal method employed.

## Material and methods

### Experimental design

**Figure 1:**
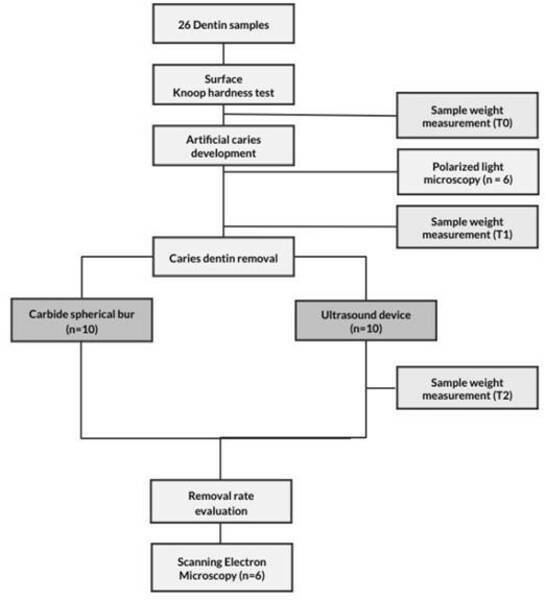
Flowchart of the study.

 The factor under evaluation was the method of caries dentin removal (carbide bur at low-speed rotation or ultrasound caries removal device). The dependent variables were the removal rate evaluated by loss of mass and the morphological analyses assessed by SEM. Figure 1 shows the flowchart of the study.

### Sample preparation

Twenty (n=20) extracted bovine incisors without structure defects and/or enamel cracks visible on stereomicroscope under 10x magnification (SZ61, Olympus Corporation, Tokyo, Japan) were selected. The teeth were cleaned with a pumice stone paste and stored in a 0,1% (w/v) thymol solution before the beginning of the experiments.

Enamel and dentin samples with a dimension of 4x4 mm were obtained using a precision water‑cooled cutting machine (IsoMet 1000, Buehler Ltd., Lake Bluff, IL, USA). After that, the enamel was removed using a metallographic abrasive paper (#80, Tigre S/A, Rio Claro, SP, Brazil) mounted in a politrix to obtain the bovine dentin samples with a dimension of 4x4x2 mm. After the enamel removal, the samples were polished with decreasing granulation of metallographic abrasive papers (#600, #1200 and #1500, Tigre S/A, Rio Claro, SP, Brazil).

The surface Knoop hardness of the samples was measured to select those with 38 ± 2 Knoop hardness number (KHN) for the experiments. The surface hardness was performed to standardize the surface of samples and to avoid possible bias in the sample’s structure that could interfere on the cutting capacity/resistance of the removal methods.

The samples were stored in distilled water to avoid dentin dehydration before the artificial caries induction. The pH of the water was measured once (~ 7.0).

### Development of artificial caries lesion

**Figure 2:**
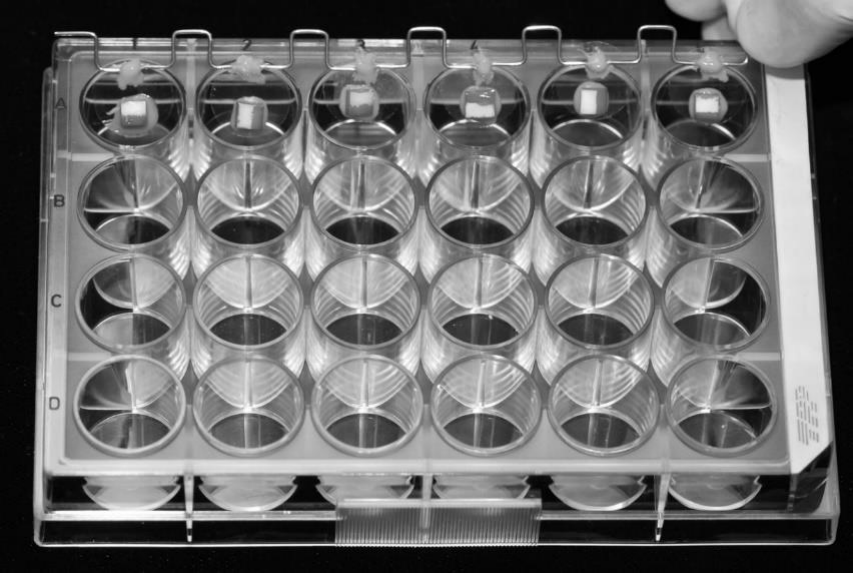
System used to maintain the samples individually suspended in a 24-well culture plate during the artificial caries lesion development.

One-half of each surface of the samples were covered with a layer of acid-resistant varnish (Colorama, CEIL Com. Exp. Ind. Ltda., São Paulo, SP, Brazil) and designated as a control group. The dentin samples were fixed under circular glass coverslips of 13 mm diameter (KASVI, São José dos Pinhais, PR, Brazil). After that, the glass coverslips containing the samples were fixed on orthodontic wire to keep the samples individually suspended within a 24-well plate to prevent the gravity action on bacterial colonization (Figure 2). The system containing the glass coverslips, the samples and orthodontic wire was autoclaved under 121ºC for 15 minutes to avoid contamination by other microorganisms and then transferred to a 24-well plate.

For the induction of artificial caries lesions, the samples were individually immersed in a bacterial suspension containing 1 mL of brain heart infusion broth (KASVI, São José dos Pinhais, PR, Brazil) and 1 mL of Lactobacilli MRS broth (Becton, Dickinson and Company, Franklin Lakes, NJ, USA) culture medium both supplemented with yeast extract 0.5 g/100 mL, glucose 1 g/100 mL and sucrose PA (pro-analysis) 2g/ 100 mL. For each 50 mL of the culture media, 5 mL of 10^8^ CFU/mL *Lactobacillus acidophilus* (ATCC #ITAL-523) and 5 mL of 10^8^ CFU/mL *Streptococcus mutans* (ATCC #25175) were added to induce bacterial colonization ^21^. The 24-well plates were incubated under microaerophilic conditions for 7 days ^22^. The culture media was refreshed every 48 hours and the pH was also measured using a pH strip indicator (Merck, Germany).

### Polarized light microscopy

 The artificial caries induction and the depth of the lesions were confirmed and evaluated by polarized light microscopy. After the induction, six dentin slices of 100 µm approximately were obtained using a microtome for metal cutting and histological processing system (EXACT Technologies Inc, Toronto, ON, Canada). The dentin slices were then fixed over a cover glass (26 x 76mm Exacta, Perfecta Lab, São Paulo, SP, Brazil) and evaluated by polarized optical light microscope (Leica DM 2500, Leica Microsystems, Wetzlar, Germany) at 10 and 20x magnifications using Leica Application Suite v3.8 software. The lesion depth was measured at the central area of each slice ^23^.

### Caries dentin removal methods

**Figure 3:**
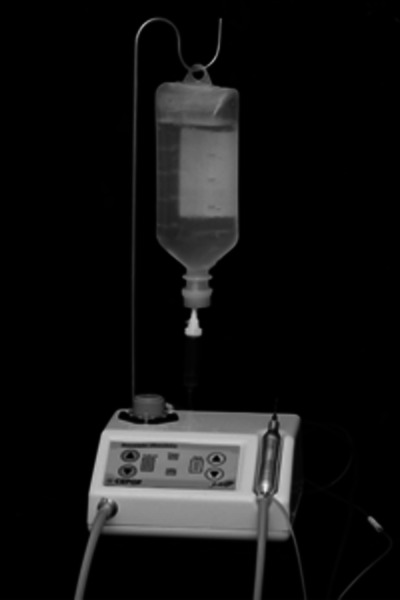
Ultrasound caries removal device.

After the artificial caries lesions development, the caries dentin removal was performed for 1 minute according to the removal methods, as follow: G1 - spherical carbide bur (FG 7, American Burrs, Palhoça, SC, Brazil) attached to a handpiece under low-speed rotation (LB100, serial number: LB1-0132680, Beltec Ind. e Com. de Equipamentos Odontológicos, Araraquara, SP, Brazil) with the following parameters: 15.000 rpm, power of 40W and frequency of 0.06 kHz (control group) and G2 - spherical diamond-coated tip (E3D, Helse Dental Technology, Santa Rosa de Viterbo, SP, Brazil) attached in a prototype of ultrasound caries-removal device (power of 30 W; frequency of 27.5 kHz) under sterile distilled water-cooled developed by the Physics Institute of São Carlos - IFSC from University of São Paulo - USP, SP, Brazil (Figure 3).

All the caries removal methods were performed with a wet dentin, in order to simulate oral conditions and under unidirectional movements. The samples were fixed in a glass plate with wax to facilitate the removal. A single calibrated operator performed all removal procedures to minimize inter-operator variability. To standardize the penetration pressure before the removal procedures, the operator pressed the handpiece coupled with the carbide bur and the ultrasound device coupled with the diamond tip against the platform of an electro-magnetic balance (BL320H, serial number: D477710288, Shimadzu Corporation, Barueri, SP, Brazil). Without looking at the balance display, the operator was instructed to apply against the platform balance the force normally used during clinical practice. The mean applied force was 103 g, which is according to the commonly force reported by the literature ^24^ ^,^ ^25^. Laboratory spotlights illuminated the operatory field, and it was considered adequate by the operator. The experiments were performed under aseptic conditions.

### Removal rate

The caries dentin removal rate was assessed by the loss of mass of each dentin sample. For this, the samples were dehydrated in an incubator at 60ºC ± 1ºC (SP-100/150, serial number: 09/16-0106, SP Labor Com. De Prod. Lab. Ltda., Presidente Prudente, SP, Brazil) for 30 minutes ^26^ and weighed using a digital precision balance (Adventurer AR2140, serial number: M254 8329080189 P, OHAUS Corporation, Parsippany, NJ, USA) at three evaluation times: T0 - before the artificial caries lesion development; T1 - before the caries dentin removal; and T2 - after the caries dentin removal. After T1 evaluation, the samples were rehydrated in sterilized phosphate buffered saline solution for 60 seconds and then the removal methods were performed.

The total loss of mass evaluation due to the artificial caries development and the caries dentin removal method employed was calculated by the difference between T0-T1 (control) and T1-T2 respectively.

### Scanning electron microscopy (SEM)

After caries removal, three samples of each group were randomly selected to SEM evaluation. The samples were dehydrated in ascending grades of ethanol (Sigma Aldrich - Merck KGaA, Darmstadt, Germany) (50% for 20 min, 60% for 20 min, 70% for 20 min, 80% for 20 min, 90% for 20 min and 100% for 60 min). The samples were mounted on aluminum stubs, sputtered with gold-palladium and then they were observed under a SEM (FEI Inspect S50, FEI Company, Hillsboro, OR, USA) at 25 kV under 200, 1000, 2000 and 5000x magnifications. The most representative areas of each sample were photographed focusing on the center of the residual dentin surface.

### Statistical analysis

The normality of the data was tested by Shapiro-Wilk test (p ≥ 0.240). After the acceptance of normal distribution, the differences within the evaluation times (T0-T1 and T1-T1) were tested. T-test for independent variables (carbide bur and ultrasound device) was applied. A p-value lower or equal than 0.05 was considered statistically significant. The analyses were carried out using the IBM^®^ SPSS Statistics^®^ version 22 (IBM, New York City, NY, USA) and GraphPad Prism (GraphPad Software, San Diego, CA, USA) software.

## Results

**Figure 4:**
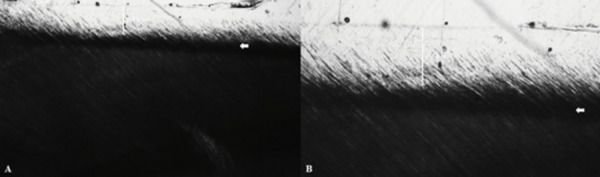
Artificial dentin caries lesion and lesion depth at 5x (A) and 10x (B) magnification obtained by polarized light microscopy images. Arrows indicate caries-affected dentin.

In order to ensure an ideal microenvironment for caries lesion development, the pH was measured before refreshing culture medium. The pH’s values obtained were around 4.0. Additionality, polarized light microscopy images showed caries dentin lesion formation with 216.6 ± 18.65 µm depth. Figure 4 shows a representative image with the lesion formation and depth (Figure 4).

**Figure 5:**
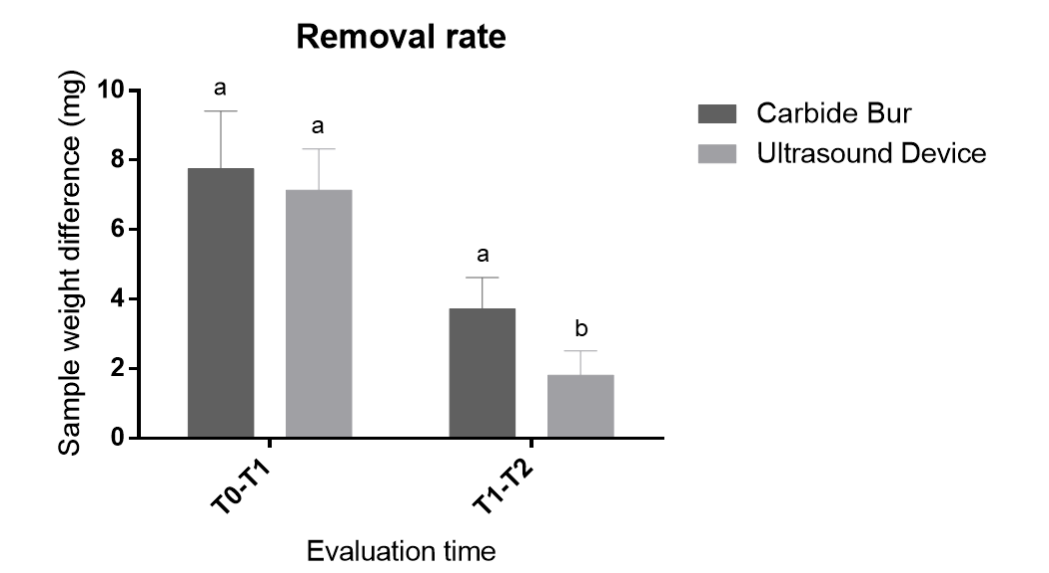
Mean and standard deviation of the samples weight difference (mg) according to each evaluation time (n = 10). T0 - before the artificial caries lesion development; T1 - before the carious dentin removal; and T2 - after the carious dentin removal.

Results for the removal rate are shown in Figure 5. No differences between the groups after the artificial caries development (baseline; T0-T1) (p = 0.569) were observed which indicate the standardization of the lesions and allowed further comparisons. After the caries removal treatments, the ultrasound device showed lower removal rate (p ≤ 0.001) than carbide bur.

**Figure 6:**
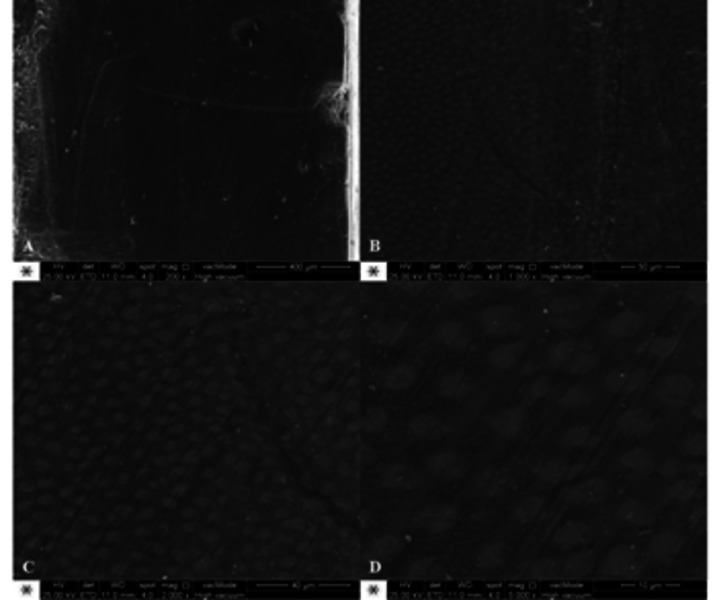
Representative SEM photomicrographs of control group (without artificial caries lesion development) at 200 (A), 1000 (B), 2000 (C) and 5000x (D) magnification.

**Figure 7:**
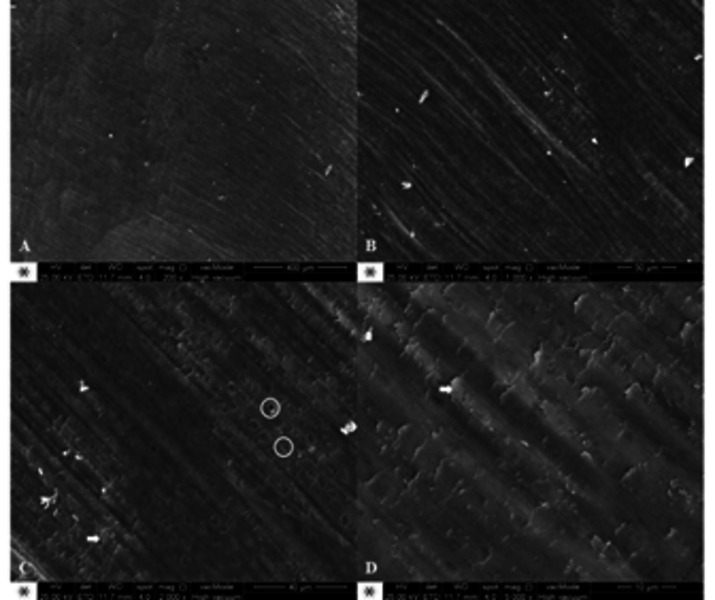
Representative SEM photomicrographs of carbide bur (G1) at 200 (A), 1000 (B), 2000 (C) and 5000x (D) magnification. Arrows indicate smear layer covering the dentin tubules. Circles indicate dentin tubules exposure.

**Figure 8:**
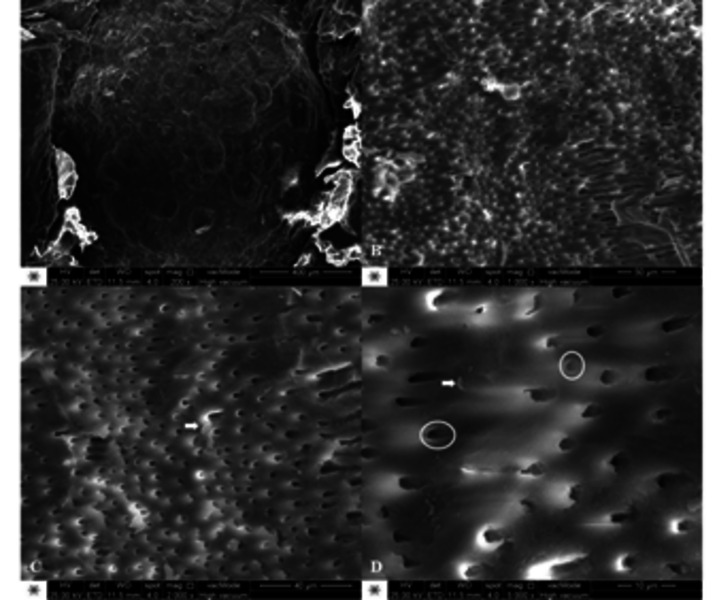
Representative SEM photomicrographs of ultrasound device group (G2) at 200 (A), 1000 (B), 2000 (C) and 5000x (D) magnification. Arrows indicate smear layer. Circles indicate dentin tubules exposure.

Representative SEM photomicrographs showed dentin surface morphology after the caries removal at 200, 1000, 2000 and 5000x magnification (Figure 6, 7 and 8). It is possible to verify different patterns of the residual dentin surface among the groups. The control images (samples covered with acid-resistant varnish to prevent artificial caries lesion development) presented sound dentin without any demineralized areas and a polished surface (Figure 6). However, the samples treated with carbide bur showed a grooved surface with considerable amount of smear layer over the dentin tubules under all magnifications and few opened tubules could be observed (Figure 7). Differently, the caries dentin removal using the ultrasound device showed a surface with no smear layer and dentin tubules are wide open. Moreover, the surface does not exhibit grooves (Figure 8). 

*Equal lower-case letters within the same evaluation time mean no statistically significant difference (p = 0.569). Different lower-case letters within the same evaluation time mean statistically significant difference (p ≤ 0.001).

## Discussion

The null hypotheses were rejected. Caries dentin removal using the ultrasound device showed lower removal rate than the carbide bur (p<0.05). In addition, SEM images highlighted that ultrasound device yields a residual dentin morphology after the caries removal quite different from conventional carbide bur.

The induction of artificial caries dentin lesions by a biological model was chosen since this method is considered more suitable to simulate natural caries lesions ^27^. Two species of bacteria (*S. mutans* and *L. acidophilus*) were used to fulfill the current paradigm, which claims that oral bacteria are part of a complex and interactive microbial community rather than a reductionist view of a single specie as the etiologic agent of dental caries ^28^. Also, these two bacteria play an important role in the development of dental caries lesions ^28^ ^,^ ^29^.

Polarized light microscopy has been used to evaluate the caries lesion depth ^30^. Then, to evaluate the removal methods in this study, the polarized light microscopy was used to show the depth of the lesion and confirm its formation. Our results showed that 7 days was enough time to induce dentin caries lesion with approximately 200 µm depth (Figure 3). This finding is according to a previous study that used the same induction time (7 days) to evaluate the influence of dentin caries tissue on microtensile bond strength after adhesive system application ^22^. Moreover, it has been reported that this time is optimal to induce caries-like lesions using *S. mutans* and *L. acidophilus* ^30^. Therefore, the samples were immersed in the bacterial suspension for 7 days. 

The pH is a crucial factor for caries lesion development. Cariogenic biofilms are acidic and hypoxic which creates an ideal microenvironment for organisms, such as streptococci and lactobacilli to grow and to accelerate caries progression ^31^. In this way, to ensure the ideal microenvironment, pH was measured at every refreshing of the medium during the caries lesion development. All pH values obtained were acidic around 4.0. Moreover, microaerophilic condition was used to create a hypoxic environment.

The current guide for caries tissue removal is preserving non-demineralized and remineralizable tissue ^32^, such as caries-affected dentin. Non-selective removal is more aggressive and may lead to pulp exposure in deep caries lesions ^32^ which has a great impact on the treatment prognosis and long-term costs. In this way, minimally invasive approaches such as selective removal and stepwise excavation are preferable rather than complete removal of the caries tissue, due to reduced risk of pulp exposure ^33^, pulpal symptoms ^34^ and postoperative pulpal complications ^35^.

Since the clinical differentiation between infected and affected-caries dentin and the subjectivity of parameters for the removal are challenges in clinical practice, dental clinicians report difficult to establish a limit for tissue removal ^36^. Therefore, novel strategies have been developed to provide a conservative management during caries removal, such as polymer burs, air abrasion, chemo-mechanical methods, lasers ^8^ ^,^ ^10^, CVD diamond burs ^37^ and ultrasound tips ^13^.

Ultrasound instruments use energy with a wave frequency of 20 kHz above human hearing ^15^ and promotes vibration. This vibration can be basically generated by the magnetic or piezoelectric method ^38^. In the magnetic method, changes in the magnetic field convert electric magnetic energy to mechanical energy. On the other hand, in the piezoelectric method, the vibration is produced by the switch of the electric charge to cause the dimension deformation of piezoceramic disks ^15^. The piezoelectric effect has some advantages that include higher efficiency of energy transfer and greater vibration from a linear motion ^15^.

The vibration mode and amplitude created by ultrasonic instruments depends on the morphology, structure design, frequency, and power supply of the devices ^39^. Additionality, the physical properties of the devices, like node and anti-node vibration characteristics, must be taken into consideration in order to achieve an appropriate structure design, with matched frequencies, to create an ideal vibration for clinical application ^15^.

Ultrasound devices can remove caries lesions by abrading the tissue with oscillating diamond-coated tips ^13^. These oscillations can provide minimally invasive cavity preparation, easy removal of caries located in hard-to-reach areas, such as proximal caries, due to specific angulate shapes of oscillating tips ^14^, low noise level ^13^, minimal thermal change and reduced pain and/or sensitivity during cavity preparation ^13^ ^,^ ^37^.

In this study, the use of ultrasound device showed to be more conservative on caries removal than conventional carbide bur. This result confirms the tendency toward the use of ultrasound energy as a tool for minimally invasive cavity preparation ^13^. In addition, the less aggressive and more gradual removal suggest that ultrasound device can be useful to guide dental clinicians regarding the point that dentin removal should be stopped and to remove infected tissue in cavity areas that requested minimal intervention and controlled removal, such as pulp wall in deep caries lesions when the risk of pulp exposure is high.

Besides the removal effectiveness, the investigation of the morphology of residual dentin is also important. After caries dentin removal, the characteristics of the substrate play an important role during the restorative procedures ^40^, since the dentin substrate and architecture may be changed by physiological aging and disease process ^18^ ^,^ ^41^. Furthermore, it has been reported that the bond strength decreases according to the degree of caries progression. Caries-infected dentin exhibit a poor prognosis related to bond strength ^42^ due to the low cohesive strength caused by low degree of mineralization and the collagen-matrix disorganization ^3^. Moreover, polymerization shrinkage is an inherent physicochemical phenomenon resulting in an interface gap when a resin-based composite is placed that allow toxic substances by microbiota invasion. To overcome this problem, it has been suggested that infected dentin should be removed completely from preparation walls but selectively from the pulpal and/or axial wall ^42^. For this, a conservative and selective removal method is required. In this way, the ultrasound device seems to be a conservative and selective method, considering the results obtained in this study.

Smear layer is an amorphous layer of organic and inorganic debris formed on the surface after mechanical cavity preparation ^43^. It is well established that smear layer can decrease dentin permeability and the bond strength ^43^ ^,^ ^44^. In this study, SEM analysis showed that G2 (ultrasound device) showed no smear layer over the dentin surface. The morphology of the residual dentin surface may be related to the oscillation of the ultrasound device. This result corroborates with a previous study that reported complete elimination of smear layer after sono-abrasion of the dentin ^45^. The surface almost free of smear layer after ultrasound removal may positively affect the bond strength of adhesive system to dentin. However, this potential advantage in terms of bond strength is adhesive system-dependent. Based on the SEM images, the ultrasound provided a residual surface more favorable to the use of etch-and-rinse adhesive systems that act removing smear layer ^46^. However, the evaluation of the resin-dentin bond strength should be performed to support this statement. Furthermore, it is important to emphasize that the removal techniques used can display several cutting profiles with different effects on dentin surface ^47^.

SEM images also display that both removal methods provide a residual dentin surface with no remaining bacteria, which infer that all the infected tissue was removed. These results are in accordance with previous studies that used SEM to evaluate residual dentin ^48^ ^,^ ^49^.

The use of the ultrasound device can improve the minimally invasive procedures in restorative dentistry. A recent systematic review claimed a need for well-structure studies to enhance the recommendation of oscillating devices, such as the ultrasound ^47^. In this way, this study provided new insights in terms of ultrasound energy for caries treatment and may encourage the technical development in this field. Also, clinical studies are always needed and essential in order to obtain more reliable findings.

According to the results obtained in this study, some conclusions can be drawn. The ultrasound device showed lower removal rate of the caries dentin lesions than carbide bur. SEM images showed that the ultrasound device provides a residual dentin surface with low amount of smear layer and open dentin tubules, which was very different that obtained by the carbide bur. Both methods exhibited a dentin surface with no remaining bacteria. In addition, the results suggest that the ultrasound device can be a minimally invasive approach for caries dentin removal, encouraging further experiments.
